# Development and Progress in Sensors and Technologies for Human Emotion Recognition

**DOI:** 10.3390/s21165554

**Published:** 2021-08-18

**Authors:** Shantanu Pal, Subhas Mukhopadhyay, Nagender Suryadevara

**Affiliations:** 1 School of Computer Science, Faculty of Science, Queensland University of Technology, Brisbane, QLD 4000, Australia; shantanu.pal@qut.edu.au; 2 School of Engineering, Faculty of Science and Engineering, Macquarie University, Sydney, NSW 2109, Australia; 3 School of Computer and Information Sciences, University of Hyderabad, Hyderabad, Telangana 500046, India; nks@uohyd.ac.in

**Keywords:** sensors, human emotion, motion analysis, wireless communications, Internet of Things, wearable sensors, physiological parameters monitoring

## Abstract

With the advancement of human-computer interaction, robotics, and especially humanoid robots, there is an increasing trend for human-to-human communications over online platforms (e.g., zoom). This has become more significant in recent years due to the Covid-19 pandemic situation. The increased use of online platforms for communication signifies the need to build efficient and more interactive human emotion recognition systems. In a human emotion recognition system, the physiological signals of human beings are collected, analyzed, and processed with the help of dedicated learning techniques and algorithms. With the proliferation of emerging technologies, e.g., the Internet of Things (IoT), future Internet, and artificial intelligence, there is a high demand for building scalable, robust, efficient, and trustworthy human recognition systems. In this paper, we present the development and progress in sensors and technologies to detect human emotions. We review the state-of-the-art sensors used for human emotion recognition and different types of activity monitoring. We present the design challenges and provide practical references of such human emotion recognition systems in the real world. Finally, we discuss the current trends in applications and explore the future research directions to address issues, e.g., scalability, security, trust, privacy, transparency, and decentralization.

## 1. Introduction

**Figure 1:**
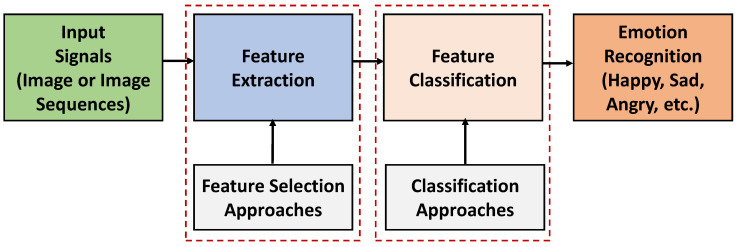
A simple process involved in the human emotion recognition system.

Emotion is significant in our daily life, and it plays a vital role in how we think, react, and behave [1]. In other words, it is a central part of decision making, problem-solving, communicating, or even negotiating in different situations [2]. Emotion can be regarded as a mental state that changes and is often characterized by physiological changes that can be seen by external physical expressions as well as observed by internal feelings. Thus, monitoring these changes is pivotal to detect and prevent a concern in an early stage, in particular, for those who have developed mental disabilities. Emotion recognition is a process that identifies human emotions. Figure 1 shows a basic process of human emotion recognition. It involves the steps from an input signal to emotion recognition. Input signals (e.g., an image or image sequences) are captured. Then they are processed based on feature extraction and feature classification approaches. Finally, the emotions are identified and detected. Emotions, e.g., happy, sad, angry, stress, etc., may vary in different places, circumstances, cultures, contexts, as well as with human personality, interests, etc. [3,4]. Emotion recognition is associated with ‘affective computing’ [5] research area that studies and develops systems to sense the emotional state of a user (using sensors) and process them using computer systems to recognize the emotions. This is fundamental in regards to the development of the multiple sensors applications where the devices are capable of taking the physical parameters of humans and translate them (the signals) to computational signals that are competent in expressing different human emotions in real-time. For instance, depending upon the mood of a person, lights in a room can change, or by detecting the facial expressions of students teaching methods can be improved. Emotion recognition has also become a new trend for e-marketing. Companies have effectively used this technology to attract more customers based on their choices for the promotion of products [6,7].

### 1.1. Motivation

With the development of sensor networks, the Internet of Things (IoT) technology, and wearable smart devices it can now be possible to detect different human emotions quickly and efficiently [8]. It has become a significant area of research when it comes to large-scale systems, in particular, related to human-machine interactions. It is argued that if the machines could understand humans’ affective emotional state, then it would be much easier and become smoother in communication [9]. For automatic recognition of emotions, various methods are employed. Some commonly used techniques include textual information, speech and body gestures, facial expressions, physiological signals. That said, this can be achieved by fabricating sensors based on needs and requirements that can contact a human body directly (i.e., invasive) or indirectly (i.e., non-invasive) when collecting the physiological parameters [10].

Among others, emotion recognition plays a crucial part in human health and the related medical procedure to detect, analyze, and determine the medical conditions of a person [11,12]. It is gaining popularity for the early detection of physiological and emotional changes for patients. It is often noted that during decision-making, users are influenced by their affective states. For example, during a happy situation, the perception remains biased at selecting happy events and it changes for negative situations accordingly, where the negative emotions may cause potential health problems [13]. Various data (i.e., physiological parameters) are collected via sensors and are transmitted to a computer system for analysis. For communication purposes, the commonly used technology is ZigBee. This helps in the transfer of data from sensors to computer systems. An application programming interface (API) is used for real-time data monitoring and management. Therefore, proper detection of human emotion can also improve wellness and fitness treatment by appropriate identification and disease diagnoses [14,15].

There have been several types of research going on with the objective to design and development of low cost and non-invasive physiological sensors that are being able to perform in real-time to capture and evaluate basic human emotions [16,17,18]. Recent market studies show that there is a huge demand for human emotion recognition systems. It is predicted that the global emotion detection and recognition market size would be USD 56.0 billion by 2024, compared to USD 21.6 billion in 2019. This trend in the increase indicates growth in the Compound Annual Growth Rate (CAGR) of 21% in the next few years [19]. Eventually, in emotion-sensing technology, the sensors for detecting human emotions will generate a high amount of data, including a user’s personally identifiable information (PII), sensitive health information as well as contextual information (e.g., location, date, and time) in many areas including law enforcement, entertainment, and surveillance and monitoring.

### 1.2. Contributions

Emotions are considered one of the key components of human social interaction. With the growing area of human-computer interaction (also known as human-machine interaction), it is important to study the need for computers to effectively understand this component in order to be perceived by users as truly effective in communication [20]. As of today, there is no complete, structured, and coherent architecture for human emotion recognition. The available architectures are system-dependent and rely on the specific feature and requirements of that system. This presents significant challenges in developing a robust, efficient, and scalable human emotion recognition system with flexible applications and services.

There are a few surveys that discuss human emotion recognition issues in general computing systems and are dedicated to serving certain conditions. For instance, literature [21] presents a survey of facial emotion recognition based on real-world user experiences in mixed reality. The core focus of this literature is to observing various emotion recognition in Augmented Reality (AR). Another proposal [22] discusses the possibility of automated emotion representation, recognition, and prediction based on Artificial Intelligence (AI) technology. The survey is mostly focused on the impact of emotion analysis and investigates the challenges of multimodal emotion detection for computers, robots, and integrated environments to establish an automated emotion recognition method. Proposal [2] presents a survey on human emotion recognition for the IoT and affective computing systems. In [23], a systematic study has been presented based on emotion recognition from facial expressions in an e-learning context.

Unlike these proposals, in this paper, we review the state-of-the-art development of sensors and technologies for human emotion recognition from a wider aspect of technology, design, methods, and applications. In other words, to gain the objectives of this paper, we review various types of emotion recognition techniques, their design criteria, choice of methods, and available applications. We also aim to provide a summary of our findings to the state of the trends in applying sensors in human emotion recognition and indicate the future possibilities of employing intelligent sensors for human emotion recognition [24].

Currently, there are different semantically incompatible areas of research that show different views on human emotion and their corresponding recognition systems. The novelty of this paper lies in a systematic and comprehensive approach to discussing the various human emotion recognition systems, technologies, applications, and their development challenges in single literature. The major contributions of the paper can be summarized as follows:

- We review the state of the art development and progress in sensors and technologies for human emotion recognition. In particular, we provide a systematic study of various types of emotion recognition techniques, methods, and available applications.
- We discuss the several challenges for human emotion recognition (including monitoring and analysis of behaviour patterns, and measurement actions) to develop a flexible and efficient human emotion recognition system at scale.
- We provide a summary of the state of the trends in applying sensors in human emotion recognition and indicate the potential of employing such intelligent sensing systems for human emotion recognition in the future.

### 1.3. Methodology

To examine the state-of-the-art human emotion recognition and their relevant applications, we include the comparable papers published in a wider period of time, which are relevant to our present review. In addition, we emphasize and cite the other publications that we find are also applicable and have a close correlation to this review. A range of venues is considered to get a diverse range of aspects. This includes journal papers, conference/workshop/symposium papers, book chapters, and papers from multiple disciplinary repositories (e.g., technical reports, arXiv copies, etc.).

In the papers, that are included, we mostly search the keywords of human emotion, emotion recognition, wearable sensors, robotic sensors, motion analysis, emotion category, etc. in their abstract. Then we assessed the papers by reading, whether the paper describes an architecture, provides a survey, examines different human emotions techniques, etc. Of the 300 papers we examined, we find 170 papers are relevant to our research motivation. Then each paper is examined and tested against the key objective of the paper (i.e., development and progress towards human emotion recognition). We use Thompson Routers, ACM Computing Classification System, and Google Scholar.

### 1.4. Organization and Roadmap

The rest of the paper is organized as follows. In Section 2, we present the fundamental of human emotions and discuss the classification of basic human emotions. In Section 3, we present a review of widely used sensors for human emotion recognition. In Section 4, we provide a detailed discussion on different activity monitoring and their methodologies employed in various human emotion recognition systems. In Section 5, we discuss design challenges and different applications associated with some real-world applications followed by current trends and future directions on human emotion recognition in Section 6. Finally, in Section 7 we conclude the paper.

## 2. Human Emotion Recognition

In daily life, human beings encounter various conditions and events that generated different stimuli that interfere with their emotions. Research shows that human emotions are strongly associated with the cognitive judgment that direly impacts social and cultural behavior and communication [25]. There have been various attempts made to classify the emotions and categorize them based on different parameters e.g., mood, feeling, and affect [26,27,28,29,30]. In [31], Ekman classifies human emotion based on facial expressions. Six fundamental emotions are recorded they are happiness, sadness, fear, surprise, anger, and disgust. In [32], Ekman further extends his findings and added more eleven emotions including contempt, relief, satisfaction, shame, etc. Unlike Ekman, AlMejrad [13] classifies three types of human emotions based on brain wave signals, namely, motivational (e.g., hunger, pain, mood, etc.), basic (e.g., happy, sad, fear, etc.), and self-conscious (e.g., shame, embarrassment, pride, etc). Studies, e.g., [33,34] show that emotion is an integral part of decision making in everyday life. Similarly, research presented in [35] indicates that emotions are significant to make wider attention and create cognitive, physical, and social resources. This in turn help to enhance the physical, social, and intellectual skills of human beings. In [36], Feidakis et al. present a classification of emotions based on a proposed e-learning model. In total, 66 emotions have been classified. They are assorted into two groups, namely basic emotions and secondary emotions. The former (i.e., basic emotion) consists of 10 emotions, e.g., anger, joy, etc.) and the latter (i.e., secondary emotions) consists of the remaining 56 emotions. Other studies classify emotions based on voice [37] and physiological signals [38].

It can be seen that emotions contribute significantly to generating actions and their efficient execution and control. Therefore, studying human emotions and their efficient recognition (how they react) and monitoring (how these reactions affect a user physically and mentally) becomes an important issue.

## 3. Sensors for Human Emotion Recognition

**Figure 2:**
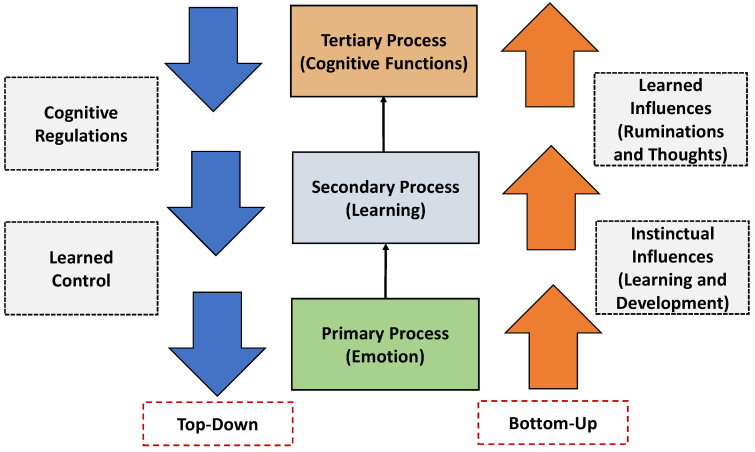
Studying human emotions to generate appropriate decisions based on control and cognitive regulations (top-down and bottom-up).

Recognizing human emotion is considered a fascinating task for data scientists. Studying human emotion needs appropriate sensors to deploy for collecting the right data. These sensors are generally used for automatic emotion collection, recognition, and making intelligent decisions. The key central nervous system (CNS) emotional-affective processes are, (i) Primary-process, (ii) secondary-process, and (iii) tertiary-process [39]. In Figure 2, we show these three processes related to collect human emotions and make decisions based on the collected data. In the first process (referred to as, primary process) emotions are collected by sensors. In the second process (referred to as, the secondary process), appropriate learning technology (and memory) is used for the collected data. Finally, in the last process (referred to as, tertiary process) appropriate decisions are taken based on the higher cognitive functions. Therefore, the choice of appropriate sensors is significant. In this section, we discuss different sensors widely used for human emotion recognition. Sensors are integral parts for sensing various emotions from human beings and transfer them into the database for further processing and results, often happens autonomously [40]. With the rapid improvement of sensing technologies and different IoT-enabled wearable and flexible sensors, there is an improvement towards the sensing performance, accuracy in measurement, light-weight in carrying capacity and efficiency in the obtained results [41,42].

Emotion recognition using text is widely used when it comes especially for human machine interaction. In this case, textual information e.g., books, newspapers, contents of different websites, etc. are taken into consideration as a rich source of content for human emotion detection. Cameras are sued for performance monitoring by detecting the facial expressions of the individuals [43,44]. Similarly, facial emotion recognition is an important part that helps to determine human emotion using different visual communication systems [45]. Cameras are, in general, used to detecting these emotions. In addition, robots are used to communicate with human beings using AI technology. It helps to capture both logical and emotional information. Commonly, this technique includes various movements of the cheek, chin, eyes, wrinkles and mouth [46,47,48].

Another widely used technique is speech [49]. It contains information not only limited to what the person is saying but also holds the information of the speaker including their emotions, real-time interactions, and various meanings [50]. It has great potential to recognize human emotion in a human machine interaction setting both for communication and interaction. The practical applicability is vast, for instance, in call centers to analyze the customers’ needs and the given feedbacks, in specific, the kind of emotions an individual transmits [51]. The next common physiological parameter that is considered for human emotion recognition is body movements and gestures [52,53,54]. Emotions can be featured by the whole-body posture and movement quality. Different kinds of optical sensors, ambient light sensor and fiber-optic curvature sensor, Kinect sensor, etc. can be used for this purpose [55,56]. This technique plays an important role in detecting the movements of the aged or patients in home care (i.e., monitoring health status remotely). Early detection of movements and unusual activities can help to determine the seriousness of a patient, e.g., fall, or unattended in a place for a long time [57]. It is reported that it gained mush efficiency in monitoring the movement and activity of patients suffering from chronic movement disorder issues, for example, patient sufferings from Parkinson’s disease [58]. In other words, it helps in continuous physiological monitoring that reduces human intervention but at the same time increases the efficiency of a patient’s wellbeing.

Human emotion recognition using biosensors are well adopted for both human to human and human to machine interactions [59,60]. Fundamentally, it has the advantage of direct monitoring of physiological parameters controlled by the autonomous nervous system which is affected by emotions. These sensors collect signals of different body parts, e.g., heart, skin, brain etc [61,62]. There are a number of advantages to using biosensors as a means of recognizing human emotion. These sensors are getting smaller in size so easily be fitted with any wearable device. Light weight in nature that supports the basic integration of technologies for the IoT area. Moreover, the production cost of these sensors is getting relatively less which attacks more market space for the common users [63,64].

Different physiological signals (can also be known as biosignals) are monitored and measured and measured using these biosensors. For instance, electromyography (EMG), which refers to the muscle activity which is measured by the muscle response to a nerve’s stimulation of the certain muscle (i.e., the compulsion of motor neurons) and recording the electrical activity, also known as the galvanic skin response (GSR) [65]. Sensors also collect signals from electrodermal activity that refers to the measurement of skin conductivity, which increases if the skin is sweaty [66]. It sees the changes in resistance of the skin to a small electrical current. Skin temperature also helps to detect muscle strength by measuring the temperature on the surface of the skin. Sensors attached to a person can detect a certain abnormality in skin temperature and human emotion is therefore vary based on the observed reactions e.g., calm or excited [67]. Sensors are also used to determine blood volume pulse (BVP) that indicates the volume of blood that is currently running through the vessels. A photoplethysmograph (PPG) composed of a light source and photosensor is typically attached to the person’s skin, is used to determine the level of BVP by recordings the motion and pressure artefacts [68]. Sensors are also used to recognize human emotion by measuring human respiration i.e., how deep and fast a person is breathing. The sensors are typically placed with a rubber band around the chest area. Commonly, breathing patterns changes in response to changes in emotions. Various emotions e.g., anger, excitement, anxiety, happiness, etc. can be determined using such sensors [69].

**Figure 3:**
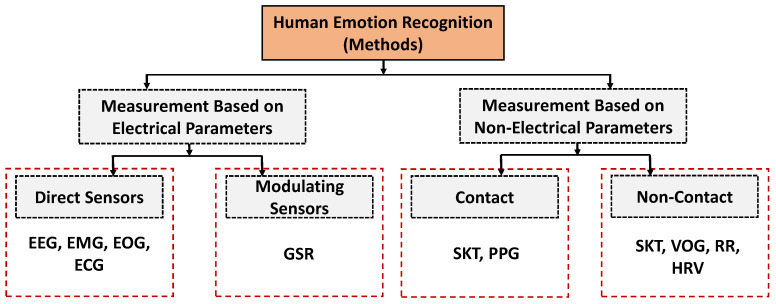
A taxonomy of different types of sensors based on the techniques, interaction, and physiological parameters.

The most commonly used biosensors for detecting human emotion is through the use of electrocardiogram (ECG) technique [70,71]. It is widely accepted to distinguish between human emotions. Sensors are placed over the chest area where the heart is situated and periodically measure the heart rates which are then used for the detention of emotions by the collected data. With the data analysis, heart rate variability (HRV) is determined which helps to reflect the state of relaxation or mental stress [72]. Sensors are also used to determine brain signals using electroencephalography (EEG) technique [73,74]. This is a popular choice to determine different emotional states by analyzing the human brain’s impulses. The EEG signals are collected using an electroencephalogram device that remains attached to a person’s scalp using adhesive-conducting gel or special headsets during the data collection process [75]. With a similar vision of EMG, eye movements are also used to determine human emotion [76]. In this case, the electrooculography (EOG) technique is used. To measure eye movement, pairs of sensors are placed either above or below the eye of a person. Sensors capture the signals that allow to determine the user’s attention for a particular object and helps to observe their subconscious behaviours [77]. With the improvements of the nanoscale devices, the use of flexible sensors shows immense potential to be utilized for healthcare, pervasive care, and industrial applications for efficient collection of data [78]. To this end, the applications of printed flexible sensors have been increased to use in human emotion recognition due to its certain advantages, e.g., low cost of fabrication, enhanced electrical and mechanical attributes, multifunctionality as well as the resolution [79,80,81]. Furthermore, the application of wearable flexible electronics [82] and affinity flexible biosensors [83] have been of great interest to collect human emotional data over the past years. With the enhancement of the printed sensors, both sensors have shown great promise for physical sensing to retrieve more insightful information. In Figure 3, we illustrate a taxonomy of different types of sensors based on the techniques, interaction, and physiological parameters.

## 4. Types of Activity Monitoring and Methodologies

There has been tremendous growth in the number of users for the different applications of human emotion recognition systems. The basic goal of such applications is to automatically collect human body parameters or electric impulses and classify a user’s emotion efficiently based on the collected information [84,85].

Different approaches and methodologies have been taken to recognize and evaluate human emotions. For this purpose, one of the most common processes is the use of natural language processing techniques. In this technique, emotions are extracted and sentiments by analyzing the input text. Proposal [86] presents a semi-automatic acquisition technique using a sentence or text for human emotion collection with the constructed emotion thesaurus. It has also used emotion-sensing and emotion computing technicians for automatic emotion detection that includes functions, e.g., syntax analysis, accidence analysis. Other design techniques, that use a robot for emotion detection based on language information have been reported in [87]. The information is extracted from words in conversations. Different communication interfaces are also used to enhance text communication that helps to understand human emotion in some specific cases [88]. Proposal [89] discusses an identification method of happiness and sadness emotions using the autonomic nervous system responses. For this purpose, two nervous signals, namely, SKT (SKin Temperature) and PPG (PhotoPlethysmoGram) were analyzed to extract a two-dimensional emotional feature vector. The signals are collected by the sensors attached to human skin. For the collection of SKT signals, the TSD200D sensor (Biopac, Goleta, CA, USA) is placed on the index finger, and, for the collection of PPG signals, the TSD200A sensor (Biopac, Goleta, CA, USA) is placed on the thumb. A cognitive-emotional model for eldercare is reported in [90]. In this model, facial expressions are collected using the Gabor filter, Local Binary Pattern algorithm (LBP), and k-Nearest Neighbor algorithm (KNN). Then using a robot these features are extracted and recognized for different variants of human emotions. The cognitive reappraisal strategy and Euclidean distance are used to obtain transition probability from one emotional state to another. With a similar vision to [88], the proposal [91] presents a new method of analyzing physiological signals with the help of the peaks (small peak and high peak) in the Electrodermal activity (EDA) signal to capture human emotion. An Empatica E4 smartwatch is used for the experiment that collects EDA, SKT, and HR values.

**Figure 4:**
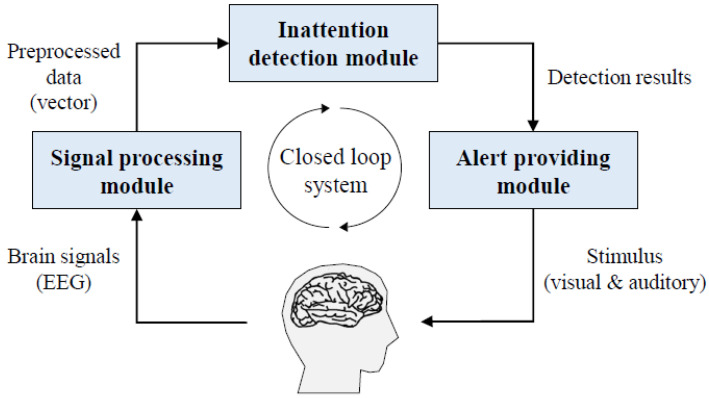
EEG-based Brian Computer Interaction Process (BCI) to collect human meotion [92].

We noted earlier that EEG is one of the popular choices for use in emotion recognition. In Figure 4, we depict the process of EEG based human emotion recognition discussed in [92]. Several approaches have used EEG for emotion recognition [93,94]. For instance, the proposal [95] uses EEG to collect peripheral signals. In the proposed scheme, three methodologies have been used. First, both the peripheral physiological features and the EEG features are extracted. Second, using canonical correlation analysis (CCA), a new physiological feature space is used. Finally, different emotional labels are created to map the peripheral physiological features using a support vector machine (SVM). Note, both the peripheral physiological signals and EEG signals play an important part in this case. To avoid the noise, for EEG signals a lower cutoff frequency of 0.3 Hz and a higher cutoff frequency of 45 Hz is used. In proposal [96], SVM and gaussian process (GP) models are sued to determine music emotion estimation. The motivation of this study is to develop a comparison in the performance between SVM and GP models to the music genre and emotion recognition tasks. Another proposal [97] presents a real-time emotion recognition system based on different human emotions collected from EEG signals. Through the analysis of brain waves, the system can recognize similar discrete emotions that are closer to the valence-arousal coordinate space. The system includes six fundamental modules, e.g., emotion elicitation, EEG data acquisition, data preprocessing, feature extraction, emotion classification, and human-machine interface. A standard database consists of 16 video clips is used for identifying an individual’s emotional states.

Earlier, we noted that there is a strong correlation between the human emotional state and physiological responses [98,99]. Therefore, collecting appropriate human emotions and their efficient management is important to understand and classify human emotions. A commonly used mechanism for human emotion detection is the use of machine learning (ML) technology. For example, the proposal [100] uses six different types of machine learning algorithms to identity two negative emotions, namely, sadness and disgust. The sensors are used to collect the physiological signals (e.g., EDA, SKT, ECG, and PPG). The results are analyzed based on the preferred algorithm to understand the driver’s emotion and driving pattern. Note, negative emotions are taken into consideration because it is primarily responsible for a gradual declination or the ability of a person’s normal thinking process. Proposal [101], discusses the use of the cross-corpus evaluation technique to examine performances in data analysis obtained from different sensors and provide a realistic view of obtainable performances. The cross-corpus evaluation technique is used in ML disciplines that has the benefits to automatically detect similarity among multiple databases of specific emotion labels. In this proposal, the authors used six different databases to see the similarities.

A machine-learning algorithm to categorize EEG dynamics based on the self-reported emotional states of a person when listening to certain music is discussed in [102]. The approach classifies four music-induced emotional states (e.g., joy, anger, sadness, and pleasure). The motivation of this study is to study and classify EEG feature extraction technique that is associated with EEG dynamics and music-induced emotional states. Unlike [100,102], which directly computing the emotions of each music piece to a desired state, proposal [103] presents a ranking-based emotion recognition approach that can be used for various applications in music organization and retrieval. In this, a collection of music is ranked based on emotions, and then the emotion values of each music are determined bases on the other music pieces by their relevance.

Image processing techniques are also used for detecting human emotions. This has gained much popularity in the development of virtual reality (VR), AR research areas, and computer vision industries. In [104], an image processing technique is used to help to understand the facial expression recognition system requirements (using video cameras). First, human emotions are captures based on facial expressions, and second, music is used on these emotions that enhance the mood of the users. A list of songs is used based on current emotions. Likewise [104], a similar study of human emotion recognition is reported in [105], which is based on customized music recommendations. Emotion recognition detection, recognize naturally induced musical emotions and their classification based on physiological changes in music listening has been reported in [106]. An emotion-specific multilevel dichotomous classification (EMDC) is employed to compare the performance with direct multiclass classification. Proposal [107], uses a speech emotion recognition (SER) system that captures human emotion using voice speech signals as an input. Five emotions are recognized, they are, anger, anxiety, boredom, happiness, and sadness. The system automatically detects the emotion, and then appropriate music selection is carried out from a pool of listed songs stored in a database. In the case of a large audio database, the use of the anchor models system is proposed [108]. In such a model, an emotion class is classified by measuring the similarity to other emotion classes. A multi-label music emotion recognition system based on the hierarchical dirichlet process mixture model (HPDMM) is reported in [109]. In this work, different components of HPDMM are shared among various models of each emotion. Linear discriminant analysis is used as a discriminant factor that captures different emotions in real-world scenarios. The proposal [110], presents a framework for human emotion in videos by transferring knowledge from heterogeneous sources (e.g., image and text). This work tries to bridge the research gap that recognizes human emotion from text sources to the video domain.

**Figure 5:**
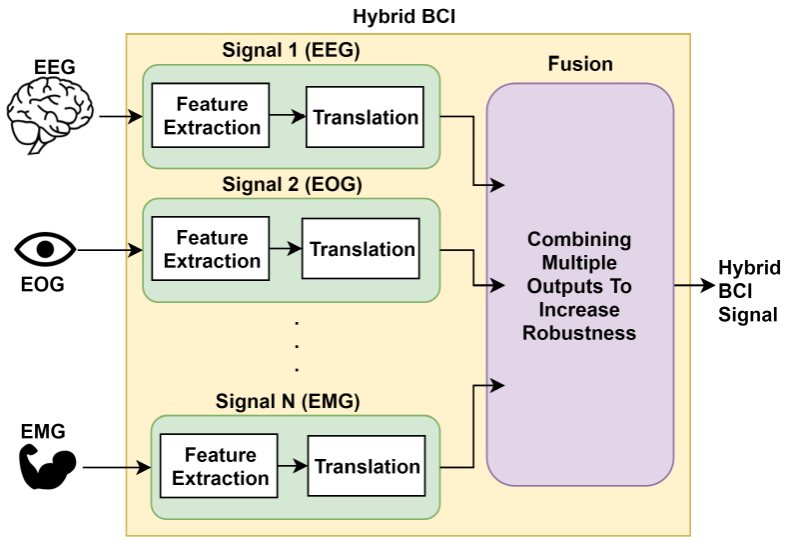
A hybrid brain-computer interface combined with EEG, EOG, and EMG signals [120].

Music is used to determine human emotion based on the noted EEG signal changes [111]. The fundamental of this is to see the brain’s processing of music in evoking different emotions. It is often argued that music acts as a direct expression of emotion from the brain waves. In Proposal [112], EEG data are collected before and after listening to music. Two kinds of music are used, preferred, and relaxing. Then the changes in emotion are measured using arousal and valence values. Likewise music, video clips are also used for human emotion recognition based on discrete emotion recognition techniques from multi-modal physiological signals, e.g., EEG, GSR, RSP, and ECG [113]. A higher-order crossings (HOC) analysis is used for emotion detection based on the EEG-based feature extraction technique has been reported in [114]. The HOC is employed for feature extraction and performs a robust classification of the different human emotional states. Another similar HOC assisted emotion recognition system using EEG signals is reported in [115]. In work [116], a technique to evaluate the emotional impact (captured by EEG signals) for each emotion is reported. An EEG-based technique is used to collect signals of different emotional expressions in the brain. The frontal brain asymmetry concept is employed to define an emotion elicitation evaluation division. A multidimensional directed information (MDI) analysis is taken into consideration that extracts various emotional measures to form an index (govern by the frontal brain asymmetry theory). This index helps to evaluate the asymmetry between the different EEG signals captured in two opposite brain hemispheres. The combination of electrocardiography (ECG) and photoplethysmography (PPG) is used to capture real-time human emotions [117]. The method is commonly known as pulse transit time (PTT). The idea of PPT is to calculate the time difference between the simulation of the heart (detected by the EEG signals) and the arrival of the corresponding blood pulse wave to a certain area, e.g., arm wrist (detected by the PPG signals). To make emotion recognition more immersive and realistic, VR scenes (e.g., using VR glasses) are used with traditional EEG-based applications [118]. A combination of EEG, EMG, and EOG signals for emotion recognition is reported in [119]. EEG signals help to recognize inner emotion, and EMG and EOG signals are used to remove artifacts. In Figure 5, we show a hybrid brain-computer interface combined with EEG, EOG, and EMG signals [120].

Note, the EEG signals of emotions vary from person to person, and there is no unique pattern for the signals. Therefore, EEG-based emotion recognition models are, in general, subject dependent. Several proposals study the need for subject independent emotion recognition based on EEG signals. It shows significance where the EEG of emotions of the subjects is not available to compose an emotion recognition model. For instance, in [121], a subject independent emotion recognition model has been reported based on variational mode decomposition (VMD) and deep neural network (DNN). VMD is used for feature extraction, and DNN is employed as the classifier for classifying emotions captured by the EEG data. Similarly, proposal [122] presents an EEG-based emotion recognition approach addressing the challenges of subject-dependencies in emotion recognition. A multi-task deep neural network model is applied to classify subject independent emotional labels.

We noted that different facial expressions are usually associated with various emotional states. In [123], an emotion detection method has been discussed that can automatically capture a smile of a person for analysis of human to human communication. Computer vision techniques are employed to capture physiological signals. The study presented in [124], considers the cultural sides of artifacts that have prominent significance in human emotion recognition. It examines the influence of language and culture on a subject’s familiarity and their perception of the overall emotion recognition. An ensemble classifier has been developed to deal with multiple languages to map and predict emotions. The diverse languages are employed for training and independently modeled the results.

The convolutional neural network (CNN) model has been proposed for human emotion recognition. It is a popular model that has been used for human emotion recognition based on a class of deep neural networks [125]. For instance, the proposal [126] presents an architecture for emotion detection that uses CNN. The proposed architecture is developed for user engagement estimation in entertainment applications. In [127], CNN is used to estimate emotions from the partially covered human face images by wearing a head-mounted display (HMD). Work presented in [128] discusses a framework that can capture emotional expressions and predict the mood of a person, perceived by other persons. In other words, the framework is able to automatically predict a person’s mood from a sequence of recognized emotions. A care-home is considered where the emotional expressions are captured using the human affective intelligence model and then the corresponding mood is determined by the experienced care-takers. CNN is also used for emotion detection in large-scale systems like the IoT. For instance, proposal [129] presents an architecture that can predict human facial expressions using a deep facial expression recognition algorithm supported by CNN. An approach to detect human emotion using both image and text has been reported in [130]. The proposed model examines the recognition of the character emotions from television drama characters which in turn will help to understand the story. To classify the images, a deep learning model (e.g., CNN) is employed to automatically identify the characters, objects, and activities. The process includes both the facial images of the television characters and the textual information that describes the situation. Seven emotional classes are considered. In [131], CNN is used to classify gray-scale images using a multi-class classifier. A single integrated module is used to detect the human face and recognize their emotions. A list of seven emotions is used for this study.

A physiological signal-based emotion recognition algorithm has been reported in [132]. The idea is to perform an efficient mapping from the discrete human emotions to a fixed pattern of physiological signals. A support vector machine (SVM) is used to automatically classify these emotions by pre-processing the physiological signals and the corresponding feature extraction. The fuzzy logic-based methodology is used for human recognition technology. For instance, a fuzzy relational approach to human emotion recognition has been proposed in [133]. The proposal uses external stimulus and studies three important regions including mouth, eyes, and eyebrows to facial expressions. Then the expressions are analyzed and segmented into individual frames of regions of interest. In other words, the fuzzy logic-based scheme is used for controlling the transition of emotion dynamics to the desired state. Several proposals have been discussed for emotion recognition in mobile platforms. For instance, the study presented in [134] discusses an emotion recognition system for mobile applications. In this, using the internal camera, the smartphone captures videos of a certain user. Selective frames are extracted from the video and generate subband images. Then based on the subband images, the local binary patterns (LBP) histogram is calculated. Finally, using a gaussian mixture model (GMM) based classifier emotions are classified.

In Table 1, we provide a summary of major methods for activity monitoring (focused human emotion recognition) and corresponding approach used (reference articles) that are discussed in this section. Overall, it can be seen that the use of different methodologies for human emotion recognition is paying much attention these days. Advancements in newly developed technologies and applications further enhance emotion recognition to an extent level for both the users and manufacturers.

## 5. Design Challenges and Applications

Sensing technology conjointly brings to life a replacement style approach that can embody many complex tasks than merely making a visual style for emotion recognition. It will combine monitoring and analysis of behaviour patterns, measurement actions, and noting facial expressions, voice intonation, and visual communication. Sensible devices are learning to assess the means of emotions they ‘perceive’ and to retort sensitively. An accurate emotion recognition process depends upon the uncertainty present in emotion recognition methods, in particular, when combining various disjoint models [135]. However, the design challenges may vary based on the system’s requirements and the designer’s choice. Challenges may come from representation learning of facial expressions, choices of sensors that can react accordingly, or even a person’s behavioural challenges to express a particular emotion [136]. Among others, one core challenge for human emotion recognition systems is present is the design of smart, intelligent, and collaborative systems which can interact in different service components of a system to provide more accurate results in real-time. Apart from that, there are electrical and non-electrical constraints that can affect the overall emotion recognition process. In Table 2, we summarize the various emotion recognition techniques based on such electrical and non-electrical constraints and provide an outline of their comparison.

The ability of everyday objects to retort to users’ emotional states may be accustomed to producing many personalized user experiences. It may be applied in instructional and diagnostic software systems, driver-less cars, personal AI, pervasive computing, sentient video games, video games, affectional toys, and different major shopper electronic devices. For instance, a refrigerator with an intrinsic feeling device might interpret a person’s mood and recommend appropriate food. Emotion and sensible home devices might offer diversion (music, videos, TV shows, or imagery) that matches the user’s current state of mind. Video games may use emotion-based training program technology to regulate game levels and problems per the player’s emotional states.

To provide an optimized human emotion recognition system by overcoming the challenges of device portability and other resource limitations (e.g., limited battery capacity, storage, and processing speed), a technical framework considering all of these aspects is significant. For our purpose, for instance, we use the *face-api.js* project [137] to see its feasibility in capturing human emotion. It is a promising initiative to test various assumptions with the model predictions and pieces of evidence to efficient detection of human emotion. This implements an AI-based CNN to solve for face detection and recognition of faces and face landmarks. To show the application scenarios, we used *face-api.js* project. With the development of such interactive systems (e.g., the faci-api.js software), the interest in facial expression recognition using resource constraint computing devices is growing gradually, and with it, new algorithms and approaches are being developed. The recent popularization of ML made an apparent breakthrough in the research field. The research is definitely on the right path, walking together with necessary fields like psychology, sociology, and physiology.

In work [138], we developed a practical smart sensors-based human emotion recognition system. Sensors continuously monitor the heart rate, skin conductance, and skin temperature. In the system, the signals (amplified and filtered) are processed by a microcontroller (C8051 Silabs microcontroller) and for the transmission, we used ZigBee technology. Furthermore, we developed an algorithm for the automatic recognition of emotions that combines various clustering techniques. Our proposed system has the potential to extract basic emotions i.e., happiness, angry, stress, and neutral from the physiological signals. It is easy to capture data using our model and even it can be integrated with a computer mouse for efficient classification of features.

## 6. Current Trends and Future Directions

With the advancement in human computer interactions technology, digital learning platforms, e-commerce sectors, IoT and smart technologies, and other wearable technologies (including low-cost, energy-aware and portable sensors development), the proliferation to the marketplace for emotion recognition systems is becoming significant in our everyday life [139,140,141,142]. The motion-sensing technology is no more in its experimental stage, it has become a reality. It is even used to understand the mental health of a person using various available mood-tracking apps in the market place [143]. This shows its acceptance and demand in users. As discussed in Section 5, the potential use of AI for face detection and recognition of faces and face landmarks helps in age estimation and gender recognition. AI shows one of the emerging data-driven and disruptive technologies towards human behaviour recognition [144]. AI technology uses ML, deep learning and federated learning processes to execute efficient learning patterns for the machines. The convergence of these newly emerging technologies in IoTs shows demand both in industry and academia. AI also shows a significant technology that has the potential to understand human emotion efficiently in long run to produce autonomous learning and motion detection [145]. More and more AI-related technologies and application platforms are enabling efficient management from sending to analyze human emotions [146]. Government, healthcare, retail, transportation, supply chain business, and many other applications are now using such emotion recognition systems for their business. For instance, automotive vehicles can use computer vision technology that helps to monitor the driver’s emotional state [147]. The sensors are used to interact with drivers to analyze emotional states, e.g., fatigue or drowsiness generate alerts for the driver. Similarly, retail stores can use computer vision emotion AI technology that captures visitors’ moods and reactions to advertise suitable products and promotions for them. In the United States, the total retail sales were recorded at USD 5.35 trillion in 2018, and the growth is expected to reach USD 5.99 trillion by 2023. This growth in e-commerce will consequently affect the marketplace of emotion recognition technologies and their market values [148].

Emotion recognition systems are becoming more sophisticated with the development of advanced wearable and processing technologies. However, attention must be paid to the efficient collection of physiological signals from sources. Suitable emotion detection models must be chosen. For this, signal processing techniques, feature extraction methods, and relevant classifiers should be employed [149,150]. As noted above, the development of AI, ML and VR technologies are considered as part of the next-generation technologies for developing human emotion recognition technologies. The future challenges, however, to build an efficient emotion recognition must consider many areas, for instance, from the data accusation to the supervision and controlling access of these data at a secure level [151,152]. The resource capability of most of the wearable devices (typically referring to the IoT-enabled portable sensors) is limited in terms of their memory, battery, and processing power. The resource-constrained nature of the sensors must be taken into consideration for the processing and analysis of heavy-weight algorithms. Security is another significant issue in the robust development of emotion recognition systems [153,154]. Given the nature and characteristics of these resource-constrained sensors, traditional security mechanisms cannot be employed directly in such constrained devices. There is a need for rethinking the light-weight security model for these devices [155]. That said, the authorized users must be given access to the resources, and at the same time, unauthorized access must be denied avoiding information leakage [156].

Human emotion recognition systems may deal with large volumes of data. It is noted that the process of human emotion recognition deals with personal information including health, location, and other highly confidential information. It is even more significant when considering large-scale IoT systems and their association with different human emotion recognition systems for securing access control and the delegation of access rights at scale [157,158,159]. Therefore, the protection of a user’s privacy is another challenge [154,160]. Furthermore, digital identity and identity management are two other important issues that must be taken care of concerning the dynamic nature and scale of the number of devices, applications, and associated services in a large-scale emotion recognition system [154,161,162]. The challenge also is to address the resource-constraint nature of the IoT devices e.g., battery capacity, processing speed, and memory capacity to capture, store and analyze effective human emotions [163,164,165,166]. Other notable challenges are the centralized control and trust issues while using such technologies for large scale systems like the IoT. To overcome these challenges, decentralized AI technology supported by blockchain is an alternative [167]. Blockchain is a tamper-evident, shared, and distributed digital ledger of transactions for crypto-currency systems that do not depends upon a trusted third party for data processing. In other words, instead of having a central ledger with data of the whole system, in a blockchain, every block contains all the necessary data [168]. This enhances the concept of the distributed ledger rather than simply creating a centralized one. As a revolutionary technology, the use of blockchain is beneficial in IoT as it overcomes the limitations of a centralized system for storing information. There have been various proposals that discuss the future perspective towards the use of blockchain in human emotion recognition to improve scalability, privacy, security, availability, and interoperability of data [169,170]. In this study, we show the different techniques and their application to human emotion recognition. In the future, we plan to conduct a more empirical study based on the techniques related to machine and deep learning technologies. Another avenue to work, more specifically, in the field of smart healthcare systems where detection of human emotion and their appropriate diagnosis are critical. Note, our review is limited to the existing methods and techniques to capture human emotions mostly based on the human point of view (i.e., various human body parameters collected through sensors). We also plan to study in more detail in the automatic collection, processing, evaluation of human emotions with minimal human interventions.

## 7. Conclusions

In this paper, we have presented a review of the development and progress in human emotion recognition systems and technologies. We have provided a detailed discussion on the various available mechanisms in the context of human emotion recognition comprehensively and systematically. We noted that biosensors are used widely for capturing human emotions in a more sophisticated way. We observed that there is a significant potential for Artificial Intelligence (AI) and Machine Learning (ML) technologies to contribute to next-generation human emotion technologies that can operate without any human interventions. However, there are significant challenges in integrating various systems and technologies in a decentralized way to build a robust and scalable embedded human emotion recognition system. In addition, security, privacy, trust, find-grained access control, and scalability are major concerns towards the development of an efficient human recognition monitoring system. However, new research is still required and currently ongoing in the area. It is expected that we will have new algorithms and systems in the future.

## Figures and Tables

**Table 1:** Summary of major methods for activity monitoring (focused human emotion recognition) and corresponding approach used (reference articles).

Method	Activity Monitoring	Approach (References)
SKT	Skin temperature	[89]
PPG	Heart rate monitoring	[89]
EEG	Electrophysiological signals (from brain)	[93,94,95]
EMG	Nerve’s stimulation of the muscle	[120]
ECG	Electrical signal from heart	[117]
EOG	Signals from outer retina	[120]
GSR	Signals from sweat gland activity	[113]

**Table 2:** Comparison of electrical and non-electrical constraints in human emotion recognition techniques.

Measurements Depending on Electrical Constraints		Measurements Depending on Non-Electrical Constraints	
Direct contact with sensors	Modulated sensors	Contact	Non-contact
EEG, EMG, ECG, EOG	GSR	PPG, RR, SKT	PPG
Less intrusive		More intrusive	
More usability		Less usability	
Interface for user is less		Interface for user is more	
Integrate new components		Moderate integration of components	

## Data Availability

Not applicable.
